# Durable natural killer cell responses after heterologous two-dose Ebola vaccination

**DOI:** 10.1038/s41541-021-00280-0

**Published:** 2021-01-29

**Authors:** Helen R. Wagstaffe, Giada Susannini, Rodolphe Thiébaut, Laura Richert, Yves Lévy, Viki Bockstal, Jeroen N. Stoop, Kerstin Luhn, Macaya Douoguih, Eleanor M. Riley, Christine Lacabaratz, Martin R. Goodier

**Affiliations:** 1 Department of Infection Biology, London School of Hygiene and Tropical Medicine, London, UK; 2 Univ. Bordeaux, Inserm, Bordeaux Population Health Research Center, UMR 1219; CHU Bordeaux; CIC 1401, EUCLID/F-CRIN Clinical Trials Platform, Bordeaux, France; 3 Inria SISTM Team, Talence, France; 4 Inserm U955, Vaccine Research Institute, Université Paris-Est Créteil, Hôpital Henri Mondor, Creteil, France; 5 Janssen Vaccines and Prevention, Leiden, The Netherlands; 6 Institute of Immunology and Infection Research, School of Biological Sciences, University of Edinburgh, Edinburgh, UK; 7 Present Address: Immunobiology Section, UCL Great Ormond Street Institute of Child Health, London, UK

**Keywords:** Immunology, Medical research

## Abstract

Natural killer (NK) cells are implicated among immune effectors after vaccination against viral pathogens, including Ebola virus. The two-dose heterologous Ebola virus vaccine regimen, adenovirus type 26.ZEBOV followed by modified vaccinia Ankara-BN-Filo (EBOVAC2 consortium, EU Innovative Medicines Initiative), induces NK cell activation and anti-Ebola glycoprotein (GP) antibody-dependent NK cell activation post-dose 1, which is further elevated post-dose 2. Here, in a multicentre, phase 2 clinical trial (EBL2001), we demonstrate durable ex vivo NK cell activation 180 days after dose 2, with responses enriched in CD56^bright^ NK cells. In vitro antibody-dependent responses to immobilised Ebola GP increased after dose 1, and remained elevated compared to pre-vaccination levels in serum collected 180 days later. Peak NK cell responses were observed post-dose 2 and NK cell IFN-γ responses remained significantly elevated at 180 days post-dose 2. Individual variation in NK cell responses were influenced by both anti-Ebola GP antibody concentrations and intrinsic interindividual differences in NK cell functional capacity. In summary, this study demonstrates durable NK cell responses after Ad26.ZEBOV, MVA-BN-Filo Ebola virus vaccination and could inform the immunological evaluation of future iterations of the vaccine regimen and vaccination schedules.

## Introduction

Adenovirus type 26 (Ad26).ZEBOV, modified vaccinia Ankara (MVA)-BN-Filo is a safe and immunogenic, two dose anti-Ebola vaccine regimen that recently received marketing authorisation approval under exceptional circumstances by the European Medicines Agency (EMA)^1–5^. Ebola virus infection continues to be a significant global health concern with ongoing outbreaks on the African continent^6^; the need for further understanding of protective immunity induced by currently licensed vaccines against Ebola virus infection is ever more pressing.

Ebola virus glycoprotein (GP), expressed on the surface of the mature virus particle, is a highly immunogenic antigen. Stimulation of human PBMC with Ebola GP (EBOV GP) in vitro induces the secretion of high levels of pro and anti-inflammatory cytokines including IL-10, GM-CSF, IL-1β, and TNFα and lower levels of IL-12, IL-18, and type I IFN^7–13^. We and others demonstrated a dependence on accessory cell TLR-4 engagement for the stimulatory effects of EBOV GP in vitro^7,10,12,13^. Further, we provided evidence that IL-12 and IL-18 secretion in response to EBOV GP leads to NK cell activation within whole human PBMC cultures, independent of Ebola vaccination status. However, IL-10 impairs NK cell activation (in particular IFN-γ secretion), suggesting that the mixed cytokine response to EBOV GP may limit NK cell activation^13^. Ad26.ZEBOV, MVA-BN-Filo vaccination induces a proliferative increase in less differentiated NK cells up to 21 days post-dose 2, accompanied by an increased frequency of CD25^+^ NK cells, suggesting an increased responsiveness to CD4^+^ T cell derived IL-2^13^.

Anti-Ebola antibodies induce NK cell antibody-dependent cellular cytotoxicity (ADCC) in human PBMC in vitro and contribute to protection against EVD^14–17^. Our previous studies show that serum collected post-dose 1 and post-dose 2 from Ad26.ZEBOV, MVA-BN-Filo vaccinated individuals induced significant NK cell degranulation (surface expression of CD107a), higher IFN-γ secretion and CD16 (FcγRIII) downregulation compared with baseline (pre-vaccination) serum^18^.

We performed an exploratory, post-hoc evaluation of the magnitude and durability of ex vivo NK cell phenotypic changes and in vitro EBOV GP-dependent NK cell responses in samples from a phase 2 clinical trial of the Ad26.ZEBOV, MVA-BN-Filo vaccine. The original trial incorporates three arms with varying time intervals (28, 56, or 84 days) between dose 1 (Ad26.ZEBOV) and dose 2 (MVA-BN-Filo). We demonstrate prolonged alterations in ex vivo NK cell phenotype with evidence of functional activation (CD25 expression) up to 180 days post-dose 2 after Ad26.ZEBOV, MVA-BN-Filo vaccination. Furthermore, EBOV GP specific serum antibodies from vaccinated subjects supported durable NK cell function up to 180 days after vaccination.

NK cell responses were highly variable between individuals (even in the presence of a fixed concentration of a standard post-vaccination serum) and were enriched in CD56^dim^CD57^+^ (more differentiated) NK cells, indicating an effect of both antibody concentration and individual NK cell differentiation status on antibody-driven NK cell effector function. In line with previous reports, infection with human cytomegalovirus (HCMV)—which induces the expansion of highly differentiated CD57^+^ and NKG2C^+^ NK cell populations with an “adaptive” (FcεR1γ^−^) phenotype and specialised towards antibody-dependent responses^19,20^—was a significant modifier of the ADCC response to EBOV GP.

In summary, NK cell responses may contribute to durable immune protection of target populations receiving the Ad26.ZEBOV, MVA-BN-Filo vaccine.

## Results

### NK cell phenotypic changes after Ad26.ZEBOV, MVA-BN-Filo vaccination are sustained at day 180 post-dose 2

Previously, we demonstrated NK cell activation (CD25/IL-2Ra expression) accompanied by proliferation (Ki67 expression) of less differentiated (CD56^bright^ and CD56^dim^CD57^−^) NK cell subsets up to 21 days after dose 2 of Ad26.ZEBOV, MVA-BN-Filo vaccination^13^. In this current study, we analyse the effect of Ad26.ZEBOV, MVA-BN-Filo vaccination on NK cell phenotype and activation in a multicentre, phase 2 randomised clinical trial (EBL2001) up to 180 days post-dose 2. PBMC-derived CD56^+^CD3^−^ NK cells from baseline (day 1; visit 0, which corresponds to administration of dose 1), day 29, 57, or 85 post-dose 1 (visit 1, which corresponds to administration of dose 2), 14 days post-dose 2 (visit 2) and 180 days post-dose 2 (visit 3) were analysed by flow cytometry. Ex vivo CD25 and Ki67 expression were measured within gated NK cells (gating strategy is shown in Supplementary Fig. 1a, b). Less differentiated (CD57^−^), total adaptive (NKG2C^+^ or FcεR1γ^−^ cells) and highly differentiated CD57/NKG2C and CD57/FcεR1γ defined subsets were measured within the CD56^dim^ NK cell subset (gating strategy is shown in Supplementary Fig. 1c).

Initially, data from all participants from all groups receiving active vaccination were combined for analysis (see Table 1 for participant numbers). Other than a significant difference in the frequencies of CD56^bright^ NK cells comparing baseline to 180 days post-dose 2, there was no significant difference in the frequencies of CD56^dim^, CD25^+^, or Ki67^+^ NK cells between vaccination visits (one-way ANOVA mixed effects analysis with Geisser–Greenhouse correction was used due to missing data caused by an unanticipated pause in the clinical trial) (Supplementary Fig. 2a–d). Similarly, there were no significant effects of vaccination on the frequencies of highly differentiated or adaptive NK cell subsets defined by the markers CD57, NKG2C, or FcεR1γ, either alone or in combination (Supplementary Fig. 2e–i). However, considerable variation between individuals in their NK cell profiles at baseline (visit 0) and at all subsequent visits limited the power of this analysis. We therefore analysed paired PBMC samples collected from individual vaccinees at baseline (visit 0) and day 180 post-dose 2 (visit 3) (*n* = 10).

**Table 1:** Randomised vaccination regimen and corresponding numbers of serum and PBMC samples used in this study.

Group	Vaccine schedule	Timing of samples used in this study (*n* = serum/PBMC)			
		Baseline (Visit 0)	Post-dose 1 (Visit 1)	14 days post-dose 2 (Visit 2)	180 days post-dose 2 (Visit 3)
1	Ad26^a^, MVA^b^	Day 1 (26/12)	Day 29 (27/7)	Day 43 (27/15)	Day 209 (26/11)
2	Ad26, MVA	Day 1 (26/6)	Day 57 (26/8)	Day 71 (26/19)	Day 237 (24/13)
3	Ad26, MVA	Day 1 (12/4)	Day 85 (12/5)	Day 99 (12/8)	Day 265 (12/10)

Study participants received monovalent Ad26.ZEBOV expressing the GP of the Ebola Zaire virus (Mayinga variant) followed by multivalent MVA-BN-Filo expressing the GP of the Sudan and Zaire Ebola viruses and Marburg virus together with Tai Forest virus nucleoprotein. Groups 1, 2, and 3 received Ad26.ZEBOV on day 1 and MVA-BN-Filo on day 29, 57, or 85 respectively.

^a^Adenovirus type 26.ZEBOV.

^b^Modified vaccinia Ankara-BN-Filo.

**Fig. 1:**
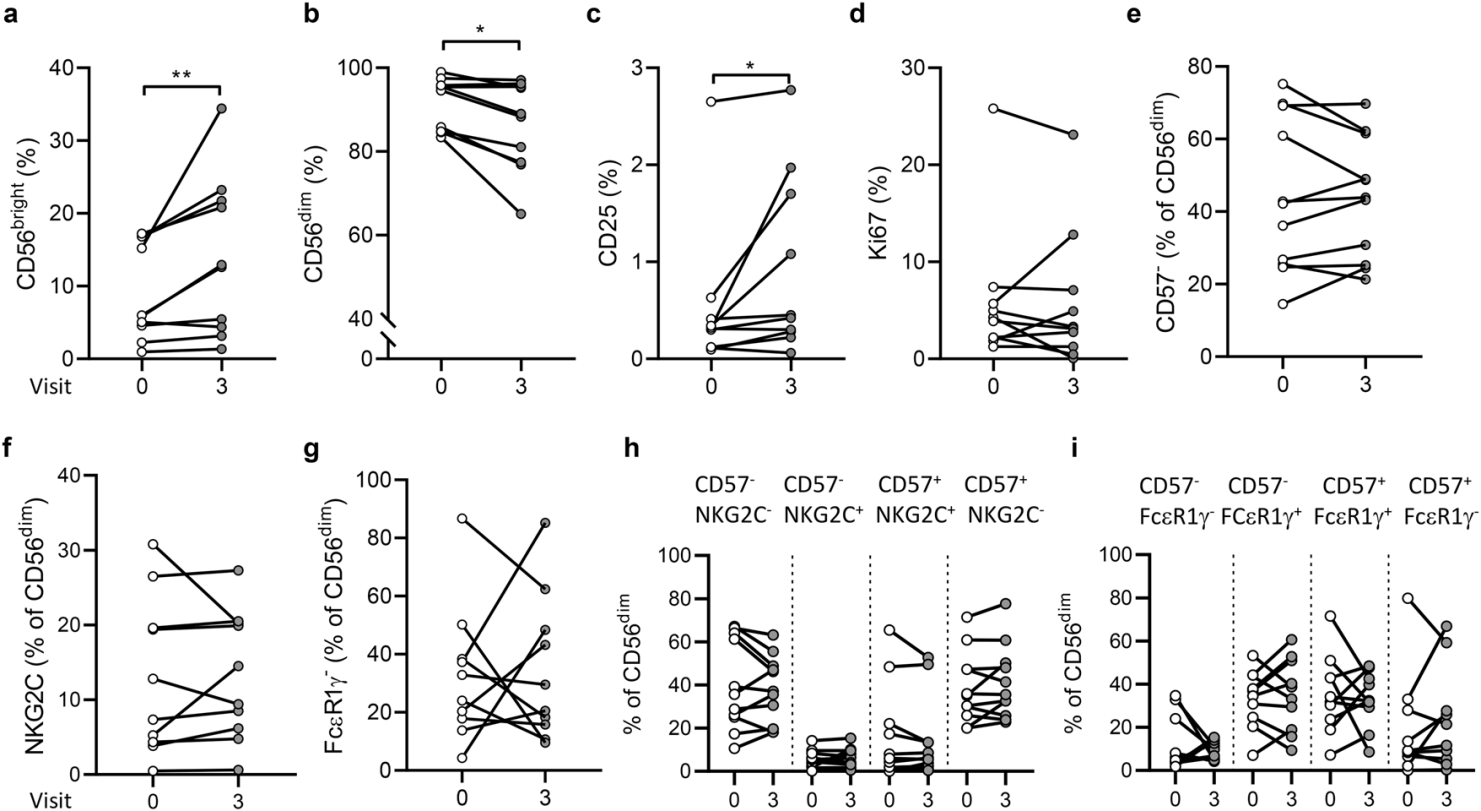
Ex vivo analysis of NK cell subsets and activation markers. Frequencies of CD56^bright^ (**a**), CD56^dim^ (**b**), CD25 (**c**), and Ki67 (**d**) expressing NK cells and frequencies of CD57^−^(**e**), NKG2C^+^(**f**), FcεR1γ^−^(**g**), CD57/NKG2C (**h**), and CD57/FcεR1γ (**i**) defined subsets within CD56^dim^ NK cells, between visit 0 (baseline) and visit 3 (180 days post-dose 2). Vaccination groups were pooled for analysis. Paired data points are shown for individual study participants as before and after plots (*n* = 10). Comparisons were made using Wilcoxon signed-rank test. *p < 0.05, **p < 0.01.

In this paired analysis (Fig. 1), CD56^bright^ NK cell frequencies were significantly elevated at 180 days post-dose 2 compared with baseline (Fig. 1a) and CD56^dim^ NK cell frequency was significantly decreased at day 180 post-dose 2 compared with baseline (Fig. 1b). The frequency of CD25^+^ NK cells was also significantly increased at day 180 post-dose 2 (Fig. 1c), although the difference in Ki67 expression did not reach statistical significance (Fig. 1d). No significant changes were observed comparing baseline and day 180 post-dose 2 frequencies of more differentiated NK cell subsets, including CD56^dim^ CD57^−^, NKG2C^+^, FcεR1γ^−^, and subsets defined by differing combinations of CD57, NKG2C, or FcεR1γ (Fig. 1e–i). In all paired comparisons between visits other than visit 0 vs. visit 3 (Supplementary Table 1), only the frequency of CD56^dim^ NK cells was significantly lower at visit 3 compared with visit 1 (*p* = 0.018), consistent with the difference compared to baseline values.

The NK cell response within each vaccination group (groups 1, 2, and 3; received Ad26.ZEBOV on day 1 and MVA-BN-Filo on days 29, 57, or 85 respectively, details of vaccine dosing intervals for each vaccine group are given in Table 1 and in the “Materials and methods” section) was analysed to test whether the interval between vaccine doses impacted on NK cell phenotype and activation (Supplementary Fig. 3a–d). A significant increase in frequency of less differentiated CD56^bright^ NK cells (Supplementary Fig. 3a), and a reciprocal decrease in representation of CD56^dim^ NK cells (Supplementary Fig. 3b) was observed across visits in group 1 (28-day interval), with a non-significant trend in group 3 (84-day interval). Groups 1 and 3 also demonstrated a trend towards increasing frequencies of CD25 expression after vaccination (Supplementary Fig. 3c), although no significant change in Ki67 expression was observed across the vaccination time course (Supplementary Fig. 3d). These data are consistent with effects on less differentiated NK cells after Ad26.ZEBOV, MVA-BN-Filo vaccination at day 180 post-dose 2 without substantial differences according to the interval between vaccine doses.

### Maintenance of post-Ad26.ZEBOV, MVA-BN-Filo vaccination-induced antibody-dependent NK cell activation at 180 days post-dose 2

We measured antibody-dependent NK cell activation (CD107a, IFN-γ induction, and downregulation of CD16; gating strategy shown in Supplementary Fig. 4), in response to baseline (visit 0), post-dose 1 (visit 1), 14 days post-dose 2 (visit 2), and 180 days post-dose 2 (visit 3) serum plus immobilised EBOV GP using a uniform responder NK cell preparation (whole PBMC from one non-vaccinated, locally-recruited blood donor).

**Fig. 2:**
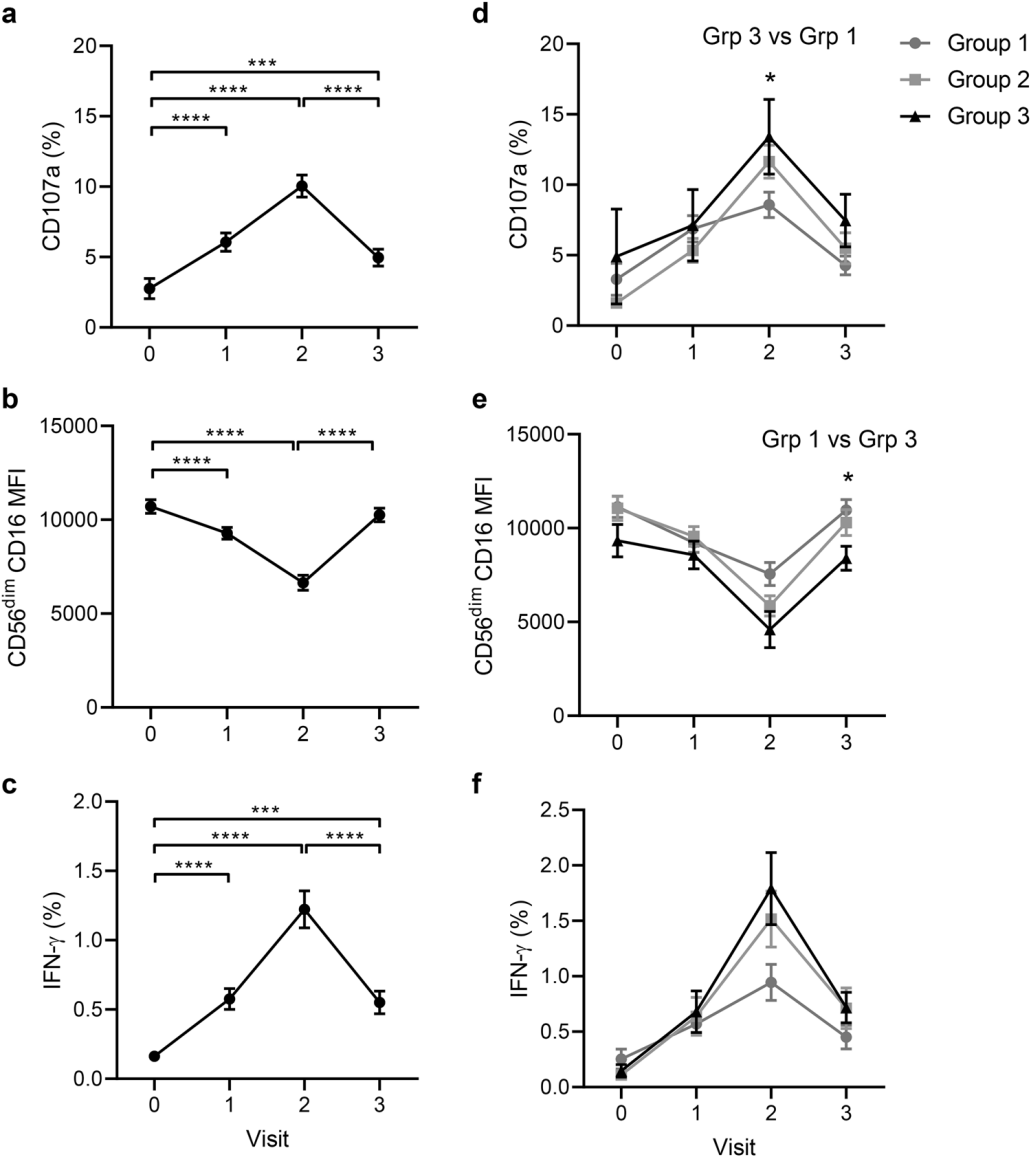
Durable antibody-dependent NK cell responses to immobilised EBOV GP. CD107a (**a**, **d**), CD16 MFI (**b**, **e**), and IFN-γ (**c**, **f**) expression among NK cells from a standard PBMC population (from a single unvaccinated donor) cultured with serum samples from individual trial donors collected pre-vaccination (visit 0), on day 29, 57 or 85 post-dose 1 (visit 1), 14 days post-dose 2 (visit 2) and 180 days post-dose 2 (visit 3). Data are shown for all vaccination groups combined (**a**–**c**) and by individual vaccination group (**d**–**f**) (see Table 1 for number of participants). Plots show mean values with standard error of the mean (s.e.m.). Across visit (**a**–**c**) and intergroup (**d**–**f**) comparisons were performed using one-way ANOVA mixed effects analysis with Geisser–Greenhouse correction. *p < 0.05, ***p < 0.001, ****p < 0.0001.

When data from all three vaccination groups were combined for analysis, there was a significant induction of CD107a expression and IFN-γ secretion, and a significant downregulation of CD16 expression (MFI) on CD56^dim^ NK cells in response to stimulation with EBOV GP in the presence of post-dose 1 (visit 1) and 14 day post-dose 2 (visit 2) serum compared with baseline serum (Fig. 2a–c). Serum from day 180 post-dose 2 (visit 3) induced a significant increase in NK cell CD107a and IFN-γ expression frequencies compared with baseline, suggesting the response is maintained at this later time point. Day 180 NK cell responses were, however, significantly lower than those observed at 14 days post-dose 2 (visit 2), suggesting a decline in antibody-driven NK cell function over time (Fig. 2a, c). Consistent with a gradual decline in antibody-mediated NK cell responses, CD16 expression returned to near baseline levels by day 180 post-dose 2, indicating more limited antibody-FcR interaction (Fig. 2b).

A trend for increasing NK cell CD107a and IFN-γ expression and CD16 downregulation in response to EBOV GP and post-vaccination serum peaking at visit 2 and reducing by visit 3 was evident in all three vaccination groups (Fig. 2d–f). Functional responses were significantly higher in the presence of visit 2 serum compared to baseline (visit 0) and remained above baseline at day 180 post-dose 2 (visit 3), in all vaccination groups (Table 2). A modest, but statistically significant, increase in the frequency of CD107a^+^ NK cells was observed in group 3 compared with group 1 at visit 2 (*p* = 0.016; Fig. 2d) and CD16 MFI was marginally lower in group 3 compared with group 1 at visit 3 (*p* = 0.046; Fig. 2e), consistent with a longer interval between the two vaccine doses promoting stronger NK cell responses. These data are consistent with the geometric mean antibody concentrations (GMC) measured for each group post-dose 2 (Group 1, GMC = 3888 units/ml, 95% CI = 2375–6364; Group 2, GMC = 8346 units/ml, 95% CI = 6207–11,224; and Group 3, GMC = 9624, 95% CI 6843–13,535).

**Table 2:** Summary statistics for anti-EBOV GP-dependent NK cell activation.

Group	Visit^a^	*p* value^b^		
		CD107a (%)	CD16 (MFI)	IFN-γ (%)
1	0 vs. 1	0.003	0.005	0.034
	0 vs. 2	<0.0001	<0.0001	<0.0001
	0 vs. 3	0.731^c^	0.978^c^	0.377^c^
	1 vs. 2	0.335^c^	0.033	0.012
	1 vs. 3	0.055^c^	0.019	0.685^c^
	2 vs. 3	0.0004	<0.0001	0.001
2	0 vs. 1	0.002	0.002	0.040
	0 vs. 2	<0.0001	<0.0001	<0.0001
	0 vs. 3	0.001	0.216^c^	0.021
	1 vs. 2	<0.0001	<0.0001	0.000
	1 vs. 3	0.998^c^	0.298^c^	0.987^c^
	2 vs. 3	<0.0001	<0.0001	0.001
3	0 vs. 1	0.639^c^	0.725^c^	0.166^c^
	0 vs. 2	0.001	<0.0001	<0.0001
	0 vs. 3	0.534^c^	0.579^c^	0.125^c^
	1 vs. 2	0.011	<0.0001	0.001
	1 vs. 3	0.998^c^	0.995^c^	0.999^c^
	2 vs. 3	0.017	<0.0001	0.001

NK cell CD107a, CD16, and IFN-γ expression in whole PBMC from a single, unvaccinated donor across the course of vaccination according to vaccination group (in relation to Fig. 2).

*IFN* interferon, *MFI* mean fluorescence intensity.

^a^Visit 0 = pre-vaccination; visit 1 = day 29 (group 1), 57 (group 2) or 85 (group 3) post-dose 1; visit 2 = 14 days post-dose 2; visit 3 = 180 days post-dose 2.

^b^One-way ANOVA mixed effects model with Geisser–Greenhouse correction. Significance was defined as *p* < 0.05.

^c^ns non-significant.

**Fig. 3:**
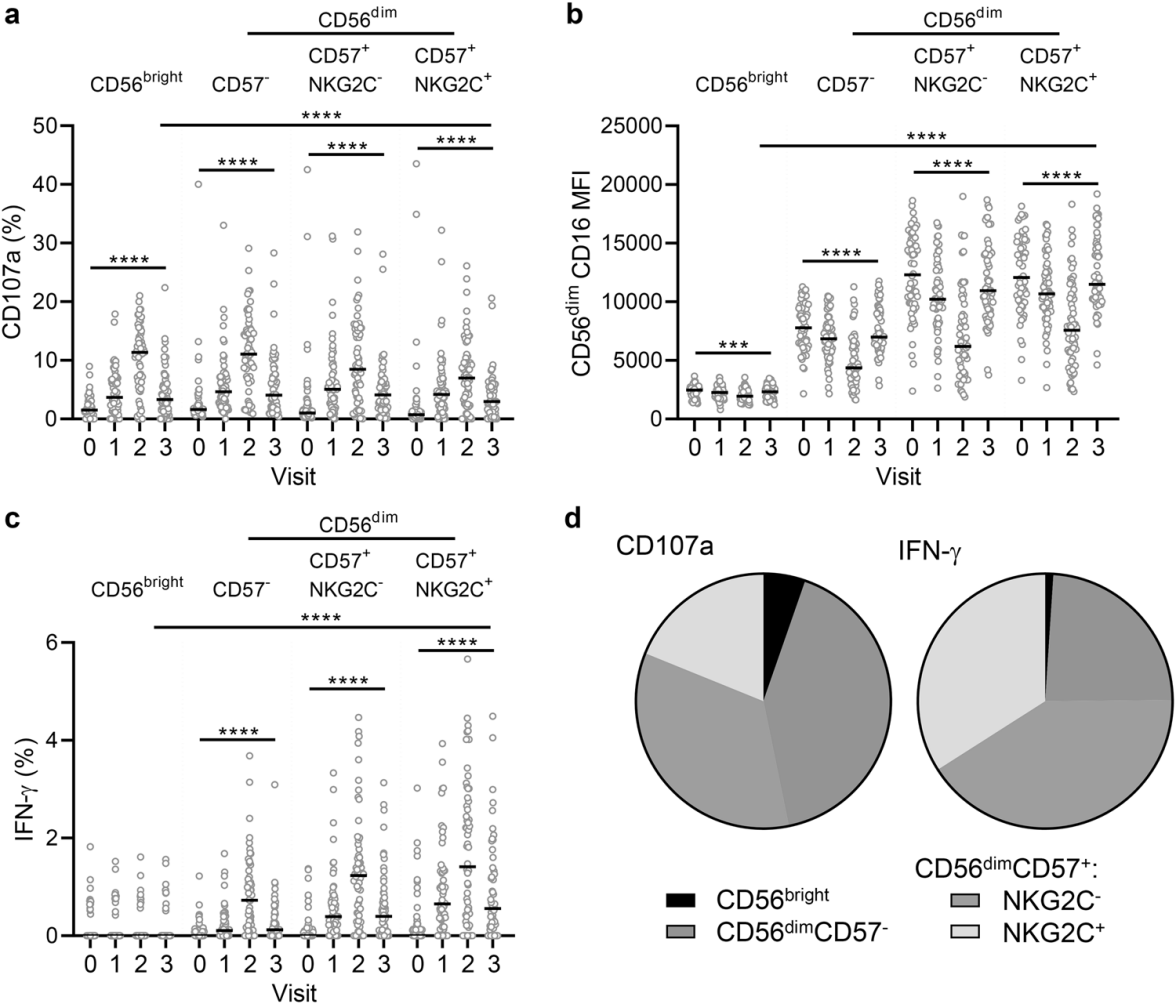
Antibody-dependent responses of different NK cells subsets. NK cells from a single unvaccinated donor were cultured with EBOV GP in the presence of serum collected from pre-vaccination (visit 0), on day 29, 57, or 85 post-dose 1 (visit 1), 14 days post-dose 2 (visit 2) and 180 days post-dose 2 (visit 3) and analysed by flow cytometry for CD107a (**a**), CD16 MFI (**b**), and IFN-γ (**c**) expression according to NK cell differentiation subset. Data are pooled for all three vaccine groups (see Table 1 for *n*). Data points are shown with bars representing median values. The proportion of total NK cell CD107a and IFN-γ expression (using visit 2 serum) attributed to each subset is shown as a pie chart (**d**), with each slice representing the median (see Table 1 for number of participants). Comparisons across visits were performed using one-way ANOVA mixed effects analysis with Geisser–Greenhouse correction and comparison between subsets was performed using and one-way ANOVA with Dunn’s correction. ****p < 0.0001.

Similar patterns of antibody-dependent NK cell responses were observed when these responses were analysed according to each NK cell differentiation subset (Fig. 3). For these experiments, cells from a single standard NK cell donor were used to minimise effects of NK cell variation and allow the effects of varying antibody responses to be elucidated. The NK cell donor was specifically selected to have a balanced distribution of NK cell differentiation subsets (CD56^bright^, 4.9%; CD56^dim^CD57^−^, 39.1%; CD57^+^NKG2C^−^, 26.3% and CD57^+^NKG2C^+^, 29.6%). CD107a expression was upregulated and peaked at visit 2 in both CD56^bright^ and CD56^dim^ subsets (Fig. 3a). Reciprocal downregulation of CD16 was also observed in all NK cell subsets analysed, with CD56^dim^CD57^+^ (NKG2C^−^ and NKG2C^+^) subsets expressing the highest levels of this Fc receptor both at baseline and after vaccination (Fig. 3b). IFN-γ upregulation was restricted to CD56^dim^ subsets (Fig. 3c), which was confirmed by back-gating analysis showing 53.2% of CD107a^+^ and 75.1% of IFN-γ^+^ NK cells were CD56^dim^CD57^+^ cells (both NKG2C^+^ and NKG2C^−^) (Fig. 3d). CD56^dim^ CD107a and IFN-γ responses were significantly higher at day 180 post-dose 2 compared with baseline, but significantly lower compared with visit 2 (Fig. 3a, c). This demonstrates a stronger antibody-dependent response within more differentiated cells, which is maintained at 180 days post-dose 2.

### Differential NK cell response between multiple vaccinated individuals in response to both autologous and pooled post-vaccination serum

We next investigated the extent to which variation in antibody concentration combined with intrinsic differences in NK cell functional capacity influenced NK cell function in response to EBOV GP in vaccinated individuals. We analysed antibody-dependent responses of NK cells (within PBMC collected at visits 0, 1, 2, and 3 from trial Ad26.ZEBOV, MVA-BN-Filo vaccinated individuals) when restimulated in vitro with EBOV GP in the presence of autologous serum collected at the same visit and, as a control, visit 2 NK cells from each subject cultured with EBOV GP and a standardised post-vaccination serum from visit 2 (pooled from all study individuals).

**Fig. 4:**
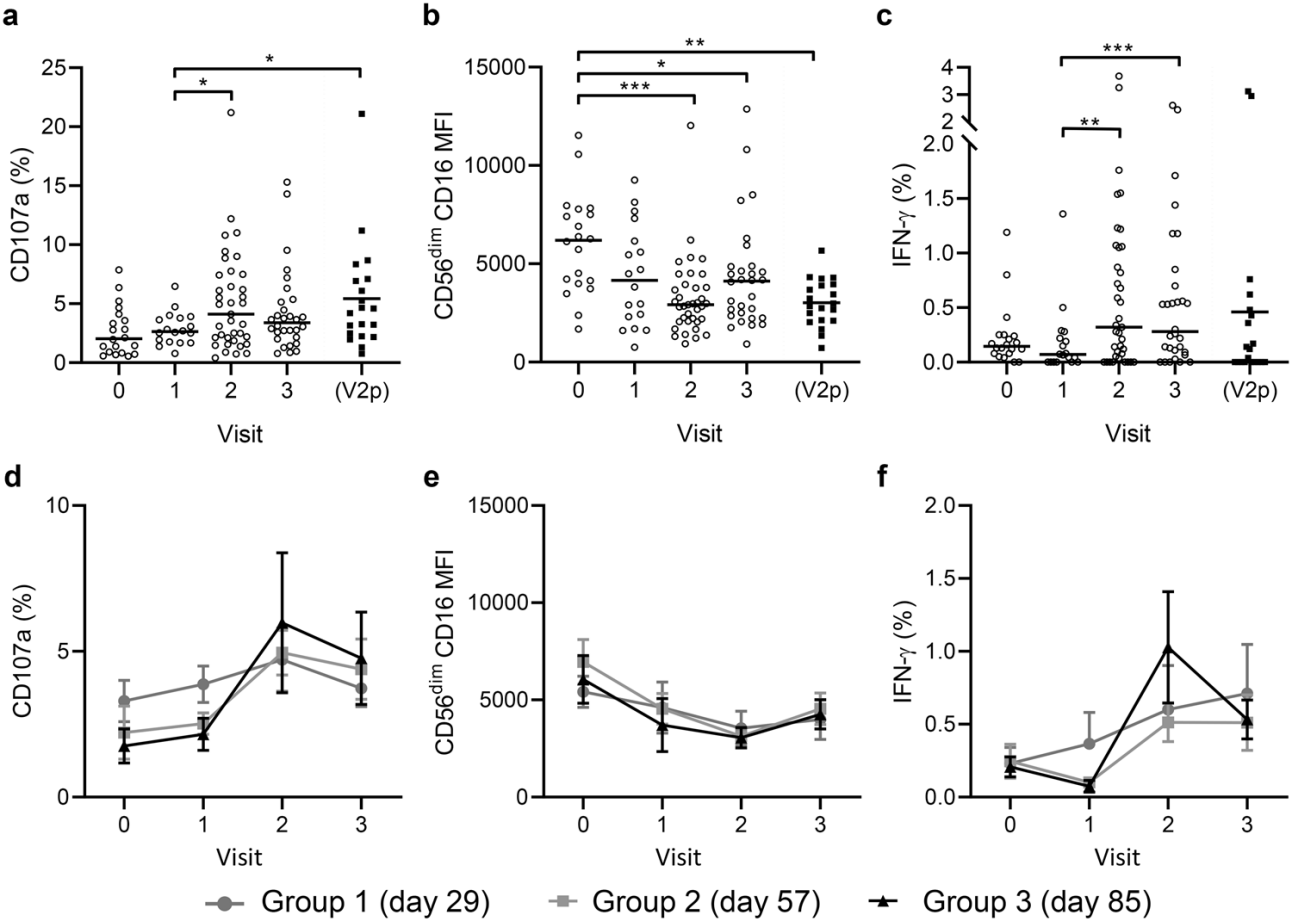
Antibody-dependent responses of NK cells from individual trial participants using their autologous pre-vaccination and post-vaccination sera. PBMCs collected pre-vaccination (visit 0), post-dose 1 (visit 1), 14 days post-dose 2 (visit 2), and 180 days post-dose 2 (visit 3) were cultured with EBOV GP in the presence of autologous serum from the same visit (or pooled visit 2 serum; V2p) and analysed by flow cytometry for CD107a (**a**, **d**), CD16 MFI (**b**, **e**), and IFN-γ (**c**, **f**) expression. Pooled data from all three vaccine groups (see Table 1 for number of participants), data points are shown with a line representing median values (**a**–**c**). Data according to individual vaccination groups are shown with mean values and s.e.m. Comparisons between visit was performed using one-way ANOVA mixed effects analysis with Geisser–Greenhouse correction. *p < 0.05, **p < 0.01, ***p < 0.001.

Consistent with the response kinetics observed for a standardised NK cell readout, median frequencies of CD107a^+^ and IFN-γ^+^ NK cells increased significantly in response to autologous visit 2 serum compared with visit 1 serum. Moreover, the frequency of IFN-γ expressing cells remained elevated (i.e., above visit 1) at visit 3 (Fig. 4a, c). Consistent with the observed changes in NK cell function after vaccination, the median MFI value for CD16 was significantly lower at visits 2 and 3 compared with baseline (Fig. 4b).

To investigate the impact of intrinsic variation within the NK cell population we also analysed individual level NK cell responses to EBOV GP plus visit 2 (peak response) serum pooled from all trial donors (visit 2 pool; V2p). In the presence of V2p serum, there was a significant induction of CD107a and IFN-γ over visit 1 and CD16 was significantly downregulated compared with baseline. Interestingly, there was no significant difference in NK cell response between visit 2 autologous serum (with varying antibody concentration) and V2p serum (constant antibody concentration) measured by one-way ANOVA (Fig. 4a–c). Moreover, the magnitude of the NK cell response of vaccinated donors was similar whether they were incubated in their own serum or the pooled V2p serum suggesting that variation was not driven by antibody concentration alone. This suggests that interdonor variation in NK cell phenotype is a driver of interindividual variation in antibody-dependent NK cell function.

When data were analysed by vaccination group, cells, and serum from group 3 tended to show stronger responses than those from group 1 and 2 (non-significant), with maximal responses seen at visit 2 (non-significant), however, low numbers of matched samples precluded robust statistical comparison between time points and groups (Fig. 4d–f).

**Fig. 5:**
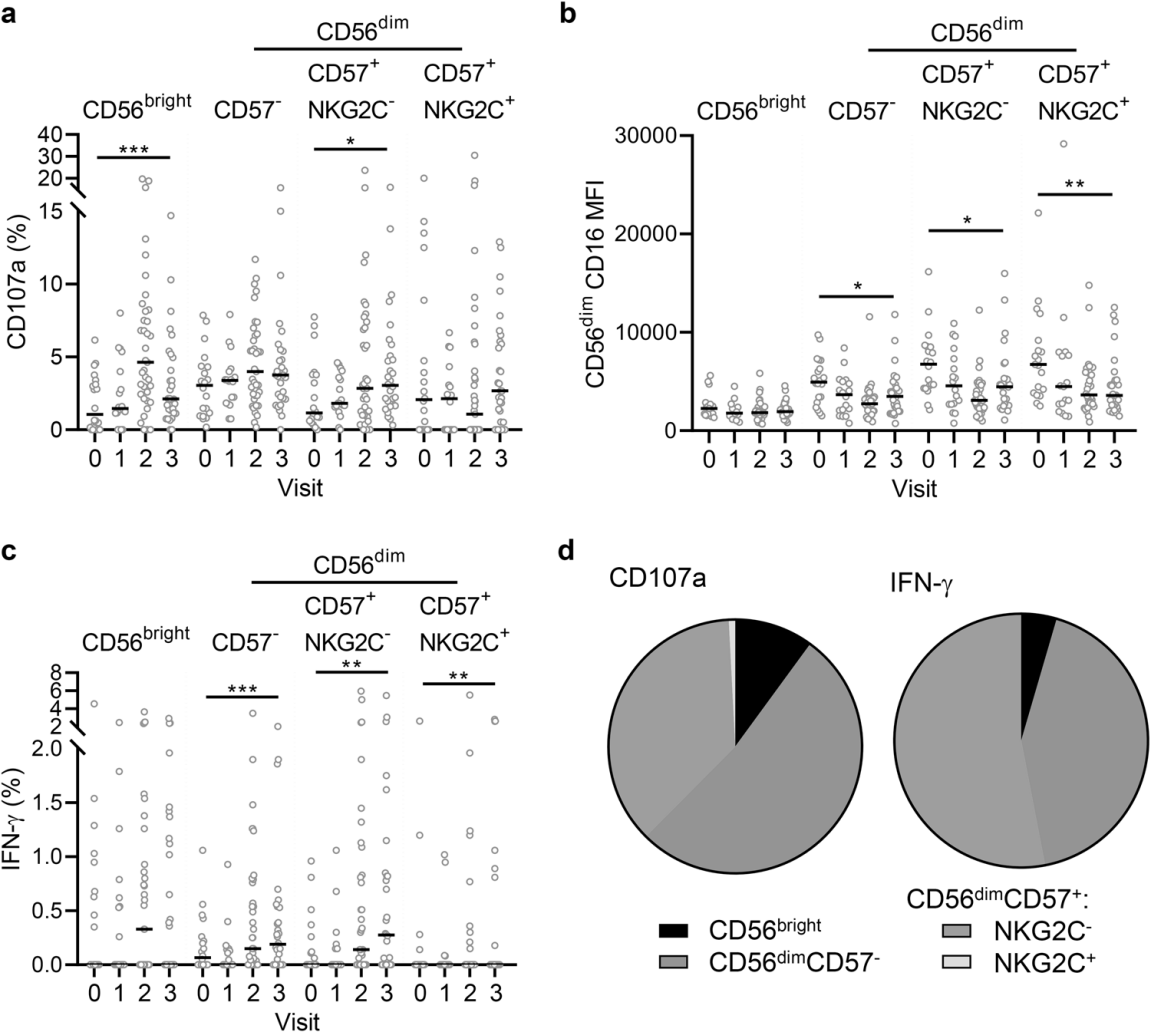
Subset distribution of antibody-dependent responses induced in multiple NK cell donors and autologous pre/post-vaccination serum. Trial participant NK cell CD107a (**a**), CD16 MFI (**b**), and IFN-γ (**c**) expression in response to autologous pre-vaccination (visit 0), post-dose 1 (visit 1), 14 days post-dose 2 (visit 2) and 180 days post-dose 2 (visit 3) serum for all vaccine arms combined were analysed according to NK cell differentiation subset. Data are pooled for all three vaccine groups (see Table 1 for number of participants), data points are shown with a line representing median values. The proportion of total NK cell CD107a and IFN-γ expression (visit 2) attributed to each subset is shown as a pie graph, with each slice representing the median (**d**). Comparisons across visits were performed using one-way ANOVA mixed effects analysis with Geisser–Greenhouse correction and comparison between subsets was performed using one-way ANOVA with Dunn’s correction.*p < 0.05, **p < 0.01, ***p < 0.001.

The frequencies of responding cells were then analysed within NK cell differentiation subsets (Fig. 5). Consistent with data obtained from the use of a single standardised NK cell readout, the majority of CD107a^+^ were CD56^dim^CD57^−^ and CD56^dim^CD57^+^ (Fig. 5a, d), the baseline expression of CD16 increasing with higher differentiation from CD56^dim^CD57^−^ to CD56^dim^CD57^+^ (NKG2C^−^ or NKG2C^+^) and being reduced to similar levels across all subsets and IFN-γ^+^ production being enriched in the most highly differentiated CD56^dim^CD57^+^ subsets, irrespective of NKG2C expression (Fig. 5c, d).

### Antibody-dependent responses of HCMV seropositive individuals with NKG2C^+^CD57^+^ NK cell expansions

**Fig. 6:**
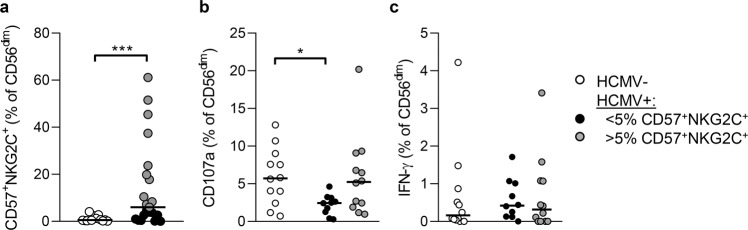
Distribution of NK cell responses in HCMV seropositive individuals. Frequencies of highly differentiated NK cells (CD56^dim^CD57^+^NKG2C^+^) in samples from HCMV seronegative (*n* = 12) and HCMV seropositive (*n* = 21) individuals post vaccine dose 2 in this cohort (**a**). CD107a (**b**) and IFN-γ (**c**) responses of CD56^dim^ NK cells HCMV seronegative (open circles) and HCMV seropositive individuals with low (<5%, black circles) and higher (>5%, grey circles) frequencies of adaptive (CD57^+^NKG2C^+^ NK cells after vaccine dose 2. Comparisons were made using Mann–Whitney *U*-test. **p < 0.01; *p < 0.01.

We observed a wide distribution of antibody-dependent NK cell responses with many individuals having little or no response to EBOV GP even within CD57^+^ and CD57^+^NKG2C^+^ subsets (Fig. 5) and considered that HCMV infection may confound this analysis^19,20^. Accordingly, HCMV seropositive individuals had significantly higher frequencies of CD56^dim^CD57^+^NKG2C^+^ NK cells post-dose 2 when compared with the seronegative individuals (Fig. 6a). Comparison of antibody dependent responses after dose 2 revealed that CD107a responses were significantly reduced in HCMV seropositive individuals with low frequencies of (<5% of CD56^dim^ NK cells) CD56^dim^CD57^+^NKG2C^+^ cells compared to HCMV seronegative individuals, and a with a reduced median response compared to HCMV seropositive individuals with higher frequencies (>5% of CD56^dim^ NK cells) of these adaptive cells (Fig. 6b). No significant difference was observed in IFN-γ production between these groups of individuals (Fig. 6c). These data suggest that individual level variation in the adaptive NK cell compartment, and differences in HCMV infection rates between populations may influence antibody dependent NK cell responses after vaccination.

### Individual level relationship between anti-Ebola glycoprotein antibody concentration and NK cell responses

Assays using a standard NK cell readout (PBMC from a single donor) to test the NK cell activating potential of post-vaccination serum from all vaccinated individuals demonstrate variation, that may be due to differences in antibody concentration. Assays using a standardised serum to test cells from multiple donors also revealed an impact of individual differences in NK cell function. In an effort to determine whether inherent variation in NK cell function or differences in post-vaccination antibody titres, or both, are drivers of inter-individual variation we looked for correlations between the NK cell response and antibody concentration. We correlated NK cell functional data from the single (unvaccinated) donor or individual trial donors in the presence of individual post-vaccination (visit 2) serum with antibody concentration.

**Fig. 7:**
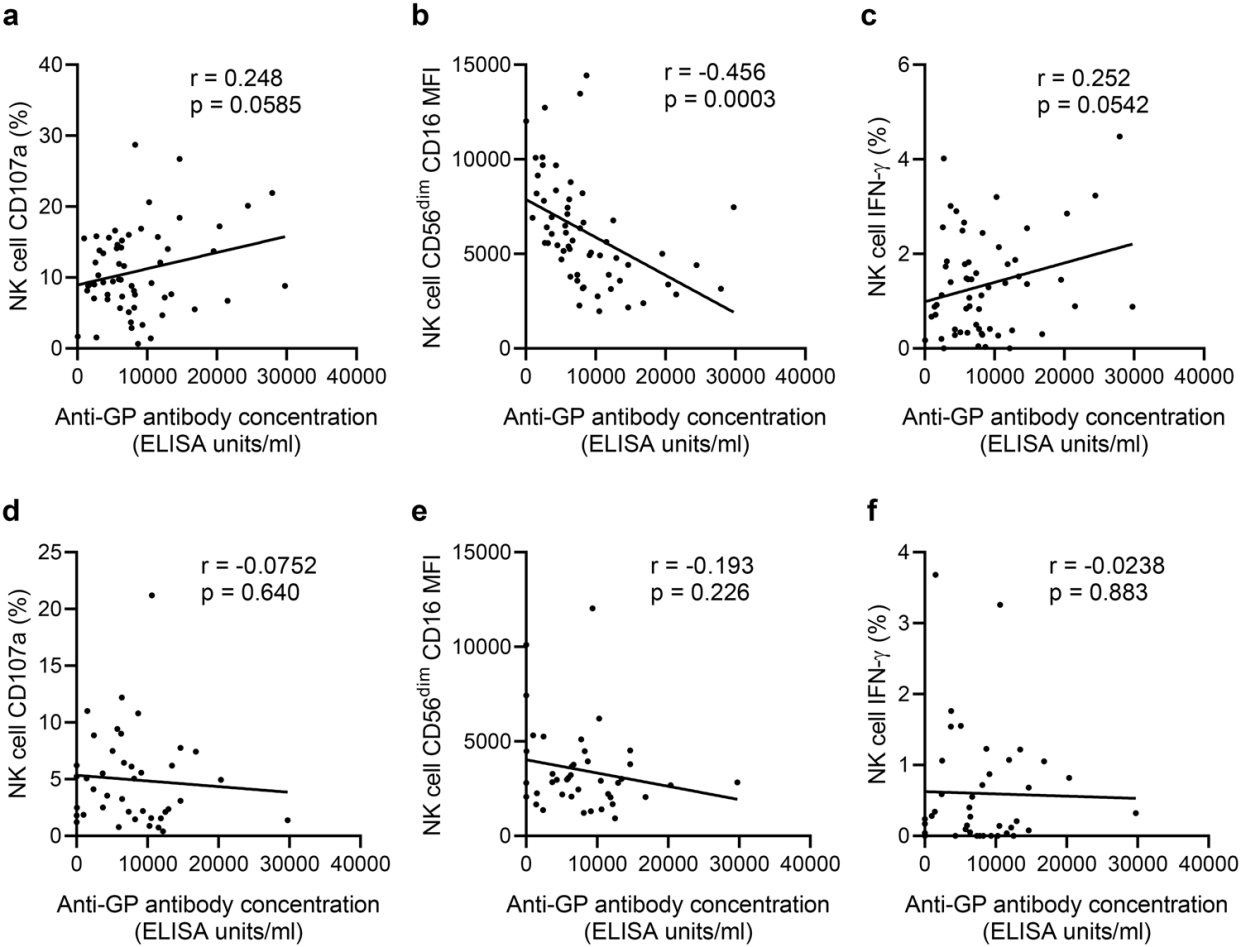
Correlation between vaccination-induced anti-GP antibody concentration and NK cell responses. PBMC from a single unvaccinated donor (**a**–**c**) or from individual study participants at visit 2 (**d**, **e**) were cultured with EBOV GP in the presence of serum collected from the trial individuals 14 days post-dose 2 (visit 2). NK cell CD107a (**a**, **d**), CD16 MFI (**b**, **e**), and IFN-γ (**c**, **f**) expression was compared with anti-GP antibody concentration (measured at visit 2) (ELISA units/ml; determined previously^38^ (see Table 1 for number of participants). Goodness-of-fit line was determined by linear regression and *r* and *p*-value analysis was performed by Pearson correlation, significance was defined as *p* < 0.05.

The NK cell function of the single non-vaccinated donor in response to EBOV GP and multiple trial serum samples from visit 2 correlated weakly with the anti-GP antibody concentration determined for each sample used (Fig. 7). CD16 MFI was significantly negatively correlated with antibody concentration (Fig. 7b), whereas CD107a and IFN-γ show non-significant, weak positive correlations (Fig. 7a, c). When each vaccination group was analysed separately, significant correlations between NK cell function and antibody concentration were observed only in group 2 (Table 3). The NK cell function of multiple trial individuals in response to EBOV GP and autologous serum from visit 2 did not correlate with antibody concentration determined for each sample when all groups were combined (Fig. 7d–f) or when analysed according to vaccination group (Table 3). These data suggest antibody concentration, the composition of the NK cell compartment and other, unidentified, NK cell-intrinsic factors may all affect antibody-induced NK cell function.

**Table 3:** Correlation between vaccination-induced anti-GP antibody concentration and NK cell responses for each vaccination group.

Group	*R*^2^ (*p* value)^a^					
	PBMC from single, unvaccinated donor			Visit 2 PBMC from individual trial donors		
	CD107a (%)	CD16 (MFI)	IFN-γ (%)	CD107a (%)	CD16 (MFI)	IFN-γ (%)
Group 1	0.0221 (0.459)^b^	0.299 (0.0031)	0.002 (0.842)^b^	0.049 (0.469)^b^	0.0075 (0.778)^b^	0.111 (0.267)^b^
Group 2	0.276 (0.0058)	0.213 (0.0176)	0.247 (0.0097)	0.116 (0.180)^b^	0.017 (0.614)^b^	0.005 (0.779)^b^
Group 3	0.0065 (0.803)^b^	0.303 (0.0635)^b^	0.0047 (0.832)^b^	0.025 (0.733)^b^	0.015 (0.796)^b^	0.008 (0.847)^b^

Correlation between NK cell CD107a, CD16, and IFN-γ expression in response to plate-bound EBOV GP plus visit 2 post-vaccination serum and anti-GP antibody concentration according to vaccination group using PBMC from a single, unvaccinated donor (left) or PBMC from individual trial donors with their autologous serum (right) (in relation to Fig. 6).

*IFN* interferon, *MFI* mean fluorescence intensity.

^a^*R*^2^ values determined by linear regression. Significance was defined as *p* < 0.05.

^b^ns non-significant.

## Discussion

Previous clinical studies of the Ad26.ZEBOV, MVA-BN-Filo vaccine regimen have shown that Ad26.ZEBOV administration followed by boosting with MVA-BN-Filo, induces high initial EBOV GP-specific antibody responses^1,2,5^. In this study, we assessed the magnitude and durability (upto 180 days post-dose 2) of changes in NK cell phenotype and function measured both ex vivo and after in vitro restimulation with EBOV GP after Ad26.ZEBOV, MVA-BN-Filo vaccination. We provide evidence of sustained enhancement of NK cell function—both ex vivo and in vitro—after Ad26.ZEBOV, MVA-BN-Filo Ebola vaccination suggesting that these cells could potentially contribute to both immediate and long-lasting vaccine-induced immunity to Ebola virus infection.

Ad26.ZEBOV, MVA-BN-Filo vaccination induced a persistent (at least 180 days post-dose 2) increase in the ex vivo proportion of CD56^bright^ NK cells but there was no significant change in the frequencies of more differentiated, adaptive subsets (CD56^dim^CD57^+^ NKG2C^+^ and FcεR1γ^−^). These data are consistent with constitutive expression by CD56^bright^ NK cell of receptors for activating cytokines, including for the myeloid lineage derived cytokines IL-12 and IL-18^21–23^, that are induced by Ebola vaccination^9,10^. The subsequent upregulation of CD25 (IL-2Rα) confers high affinity for T cell-derived IL-2, stimulating them to proliferate (and thus expression of Ki67)^24,25^. Activation and proliferation of CD56^bright^ NK cells has previously been reported as a feature of whole organism or live-attenuated vaccines^24–29^, and is line with our previous observations with this vectored vaccine including secretion of NK cell activating cytokines in supernatants of EBOV GP stimulated PBMC^13^. Recent studies, published in this journal, demonstrate dose-dependent increases in NK cell numbers and functional modulation within days of live attenuated rVSV-ZEBOV vectored vaccination^30^. The longer-term effects observed here are consistent with the rapid proliferation and relatively long lifespan of CD56^bright^ NK cells^31^. Activated CD56^bright^ NK cells migrate to secondary lymphoid tissues and, through the production of IFN-γ, contribute to priming of adaptive immune responses and recruitment of effector cells during the vaccine response^32–34^.

This study also demonstrates a major role for EBOV GP-specific antibody in activation of NK cells after Ad26.ZEBOV, MVA-BN-Filo vaccination with robust and durable antibody-dependent NK cell responses detectable for at least 180 days after the second dose in most individuals. Although not entirely consistent between the different measures of the response, a vaccination schedule in which dose 2 is given either 56 or 84 days after the first dose (rather than 28 days) tended to induce stronger and more durable antibody-dependent NK cell responses. This is in line with the benefits of delaying the second dose on immunogenicity in other trials of this two dose Ad26.ZEBOV, MVA-BN-Filo vaccine regimen^1,2,5^.

Variation between individuals in their post-vaccination antibody-dependent NK cell response reflects individual variations in antibody responses to the vaccine, in the strength of individuals’ innate cytokine responses and variations in their pre-existing NK cell populations. High resolution analysis of human peripheral blood NK cells has revealed considerable inter-individual diversity in NK cell clones that is both genetically determined^35^ and influenced by prior infection history, especially infection with human cytomegalovirus^19,20,36^. Importantly, age-related and infection-induced differentiation of human NK cells results in progressive loss of cytokine-responsive cells and concomitant accumulation of cells that are highly adapted to activation by immune complexes (termed “adaptive” CD57^+^NKG2C^+^ or CD57^+^FcεRγ^−^ NK cells), suggesting that the response to vaccination may vary with age and environmental exposures to infection, in particular human cytomegalovirus^19,20^. Indeed, we detected considerable variation in the baseline frequencies of “adaptive” CD57^+^NKG2C^+^ or CD57^+^FcεR1γ^−^ NK cell subsets among the study cohort and observed that CD57^+^NKG2C^+^ cells make an increased contribution to anti-EBOV GP antibody dependent IFN-γ responses in HCMV seropositive compared to HCMV seronegative individuals. In the context of individual variation in NK cell responses, one caveat of these studies is that they are conducted with a relatively small, highly selected, European group of heathy volunteers which may not directly reflect the responses observed in a more heterogeneous population in an Ebola endemic area. Planned future studies in African vaccination cohorts will begin to address this issue.

In summary, this study demonstrates robust, durable, antibody-dependent NK cell responses to Ad26.ZEBOV, MVA-BN-Filo Ebola virus vaccination and could inform immunological evaluation of future iterations of the vaccine regimen and vaccination schedules.

## Methods

### Study participants and samples

Eligible, healthy adult volunteers were recruited into a multi-centre, randomised, placebo-controlled, observer blind Ebola vaccine trial, held in Europe; EBL2001 (ClinicalTrials.gov Identifier NCT02416453; registered 15th April 2015, https://clinicaltrials.gov/ct2/show/NCT02416453?term=VAC52150EBL2001&draw=2&rank=2). Active vaccination comprised monovalent Ad26.ZEBOV expressing the GP of the Ebola Zaire virus (Mayinga variant) followed by multivalent MVA-BN-Filo expressing the GP of the Sudan and Zaire Ebola viruses and Marburg virus together with Tai Forest virus nucleoprotein (Janssen Vaccines and Prevention B.V., The Netherlands and Bavarian Nordic, Denmark). Groups 1, 2, and 3 received Ad26.ZEBOV on day 1 and MVA-BN-Filo on day 29, 57, or 85 respectively.

Serum and PBMC samples were collected at baseline (visit 0), post-dose 1 (day of dose 2 administration, day 29, 57, and 85 respectively; (visit 1), 14 days post-dose 2 (visit 2) and 180 days post-dose 2 (visit 3). Samples from 59 actively vaccinated donors (i.e., non-placebo arm) aged between 21 and 62 years were analysed for this study (Table 1). Additional whole blood samples were obtained from non-vaccinated, non-study volunteers from among staff and students of The London School of Hygiene & Tropical Medicine (LSHTM). The EBL2001 trial protocol and study documents were approved in France by the French national Ethics Committee “CPP Ile de France III” (reference number 3287) and the French Medicine Agency (ANSM) (reference number 150646A-61) and in the UK by the Medicines and Healthcare Products Regulatory Agency (MHRA) and National Research Ethics Service (Reference South Central—Oxford A 15/SC/0211) and the LSHTM Research Ethics Committee (reference number 14760). All participants of the EBL2001 trial provided written informed consent.

PBMC from non-vaccinated non-trial volunteers were isolated using Histopaque 1077 gradient centrifugation and cryopreserved in liquid nitrogen or used immediately. PBMC from EBL2001 volunteers from France were isolated using Leucosep tubes, cryopreserved in liquid nitrogen and shipped to LSHTM. EBOV GP-specific IgG concentration was determined by the EBOV GP Filovirus Animal Non-Clinical Group (FANG) ELISA^37^. Human cytomegalovirus (HCMV) serostatus was determined by IgG ELISA (Demeditec, Kassel, Germany) (59% of study subjects were HCMV seropositive).

### NK cell assays

Cryopreserved PBMCs were thawed, washed in RPMI 1640 supplemented with 100U/ml penicillin/streptomycin and 20 mM l-glutamine (Gibco, ThermoFisher), counted using Countess II FL Automated Cell Counter (Invitrogen, ThermoFisher) and rested for 2 h. The average cell yield after thaw was 3.0 × 10^6^ per vial. Study PBMC were either stained immediately ex vivo or were cultured in 96-well round-bottom plates in RPMI 1640 supplemented as above. 10 μg/ml purified recombinant Ebola virus GP (EBOV GP; Mayinga variant, prepared in Hek293F cells; Janssen Vaccines and Prevention B.V.) was immobilised on 96-well flat bottom tissue culture plates overnight at 4 °C. Plates were washed, blocked with 5% FCS (Gibco, ThermoFisher) in RPMI 1640 supplemented as above for 30 min and washed again. PBMC from a single, non-study donor (non-vaccinated; from fresh blood) or from vaccinated EBL2001 trial participants (cryopreserved) were washed in RPMI 1640 supplemented as above. PBMC were seeded (3 × 10^5^/well) onto the antigen-coated plates together with pre or post-vaccination serum (autologous in the case of vaccine study PBMC) or pooled visit 2 serum at 5% and incubated for 6 hours at 37 °C. GolgiPlug (Brefeldin A; 1/1000 final concentration; BD Biosciences) and GolgiStop (Monensin; 1/1500 concentration; BD Biosciences) were added for the final 3 h of culture. Cells were harvested into round-bottom plates by soaking and resuspension in cold PBS (containing 0.5% FCS, 0.05% sodium azide, and 2 mM EDTA) for staining.

### Flow cytometry

Anti-CD107a-FITC (clone H4A3; BD Biosciences) was added to the cultures (2 µl per well) for the entire culture period. Cells were stained for all other surface markers including a viability marker (Fixable Viability Stain 700; BD Biosciences) in FACS buffer (PBS, 0.5% FCS, 0.05% sodium azide and 2 mM EDTA) for 30 min in 96-well round bottom plates after blocking Fc receptors for 5 min with Fc Receptor (FcR) Blocking Reagent (Miltenyi Biotec). Cells were then washed in FACS buffer, fixed and permeabilised using Cytofix/Cytoperm Kit (BD Biosciences) or Foxp3/Transcription Factor Fixation/Permeabilisation Kit (eBiosciences) according to the manufacturer’s instructions. Cells were then stained for intracellular markers with FcR blocking for 20 min and washed again. Finally, cells were resuspended in FACS buffer and analysed using a BD LSRII flow cytometer. Cells were acquired using FACSDiva software and data were analysed using FlowJo V10 (Tree Star, Oregon, USA). FACS gates were set using unstimulated cells or FMO controls. Samples with less than 100 NK cell events were excluded from the analysis (less than 3% of samples).

The following antibodies were used: anti-CD3-V500 (clone UCHT1) (BD Biosciences), anti-CD56-BV605 (clone HCD56), anti-IFN-γ-BV785 (clone 4S.B3), anti-CD25-BV785 (clone BC96) (Biolegend, London, UK). Anti-CD16-APC (clone CB16), anti-CD57-e450 (clone TB01), Ki67-PerCP-eFluor710 (clone 20Raj1), anti-NKG2C-PE (clone 134591) (R&D systems), and anti-FcεR1γ^−^ FITC (rabbit polyclonal Ig) (Millipore, UK).

### Statistics

Statistical analysis was performed using GraphPad Prism version 7.04 (GraphPad, California, USA.). Functional responses were compared using Wilcoxon signed-rank test or one-way ANOVA mixed effects analysis with correction for multiple comparisons (detailed in figure legends). Correlation of two variables was determined using Pearson correlation analysis. Significance levels are assigned as *p = 0.05, **p = 0.01, ***p = 0.001, and ****p = 0.0001 for all tests.

### Reporting summary

Further information on research design is available in the Nature Research Reporting Summary linked to this article.

## Supplementary information

Supplementary Information

Reporting Summary

## Data Availability

The data that support the findings of this study are available from the corresponding author upon reasonable request.
