# Supplementary material for: Durable natural killer cell responses after heterologous two-dose Ebola vaccination
Source: NPJ Vaccines. 2021 Jan 29;6:19. doi: 10.1038/s41541-021-00280-0 (PMC7846750; doi:10.1038/s41541-021-00280-0)
Supplement: Supplementary file 1 — Supplementary Information [file 41541_2021_280_MOESM1_ESM.pdf]

Supplementary Table 1: Summary statistics for paired *ex vivo* data analysis.

Wilcoxon signed-rank test p value outcome between two vaccination visits (paired samples only) in the frequencies of CD56^{bright}, CD56^{dim}, CD25 and Ki67 expressing NK cells (in relation to Figure 1).

Visit [1] (n)	P value			
	**CD56^{bright}**	**CD56^{dim}**	**CD25**	**Ki67**
0 vs. 1 (n=7)	0.688 [3]	0.813 [3]	0.813 [3]	0.578 [3]
0 vs. 2 (n=14)	0.194 [3]	0.235 [3]	0.893 [3]	0.670 [3]
0 vs. 3 (n=10)	0.0059 [2]	0.0117 [2]	0.0137 [2]	0.625 [3]
1 vs. 2 (n=14)	0.197 [3]	0.122 [3]	0.0603 [3]	0.594 [3]
1 vs. 3 (n=13)	0.0681 [3]	0.0178 [2]	0.787 [3]	0.893 [3]
2 vs. 3 (n=24)	0.327 [3]	0.134 [3]	0.972 [3]	0.928 [3]

[1] Visit 0 = pre-vaccination; visit 1 = day 29 (group1), 57 (group 2) or 85 (group 3) post-dose 1; visit 2 = 14 days post-dose 2; visit 3 = 180 days post-dose 2.

[2] Wilcoxon signed-rank test. Significance was defined as $P < 0.05$.

[3] ns, non-significant.

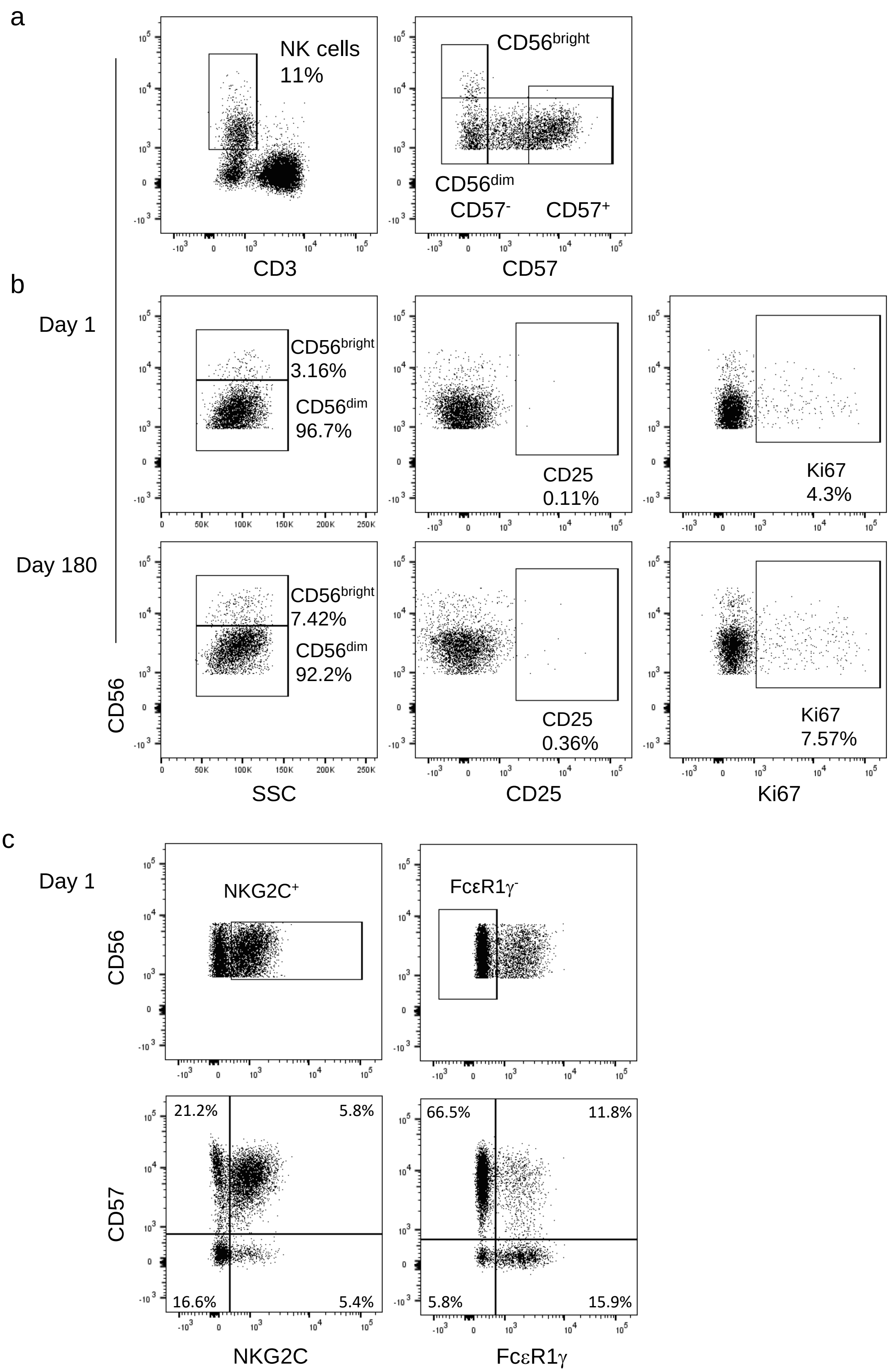

Supplementary Figure 1:
Flow cytometry gating strategy for *ex vivo* NK cell phenotype analysis.
Plots show single, live, $CD56^{+}CD3^{-}$ NK cells within whole human PBMC, and further gating into $CD56^{bright}$ and $CD56^{dim}CD57^{-/+}$ subsets (**a**). Gating of $CD56^{dim}$ and $CD56^{bight}$ NK cell subsets, $CD25^{+}$ and $Ki67^{+}$ NK cells pre-vaccination (day 1, baseline) and day 180 post-dose 2 (visit 3) (**b**). Gating of $NKG2C^{+}$ and $Fc\varepsilon R1\gamma^{-}$ NK cells after gating on the $CD56^{dim}$ subset (**c**) at day 1, plots show one representative donor.

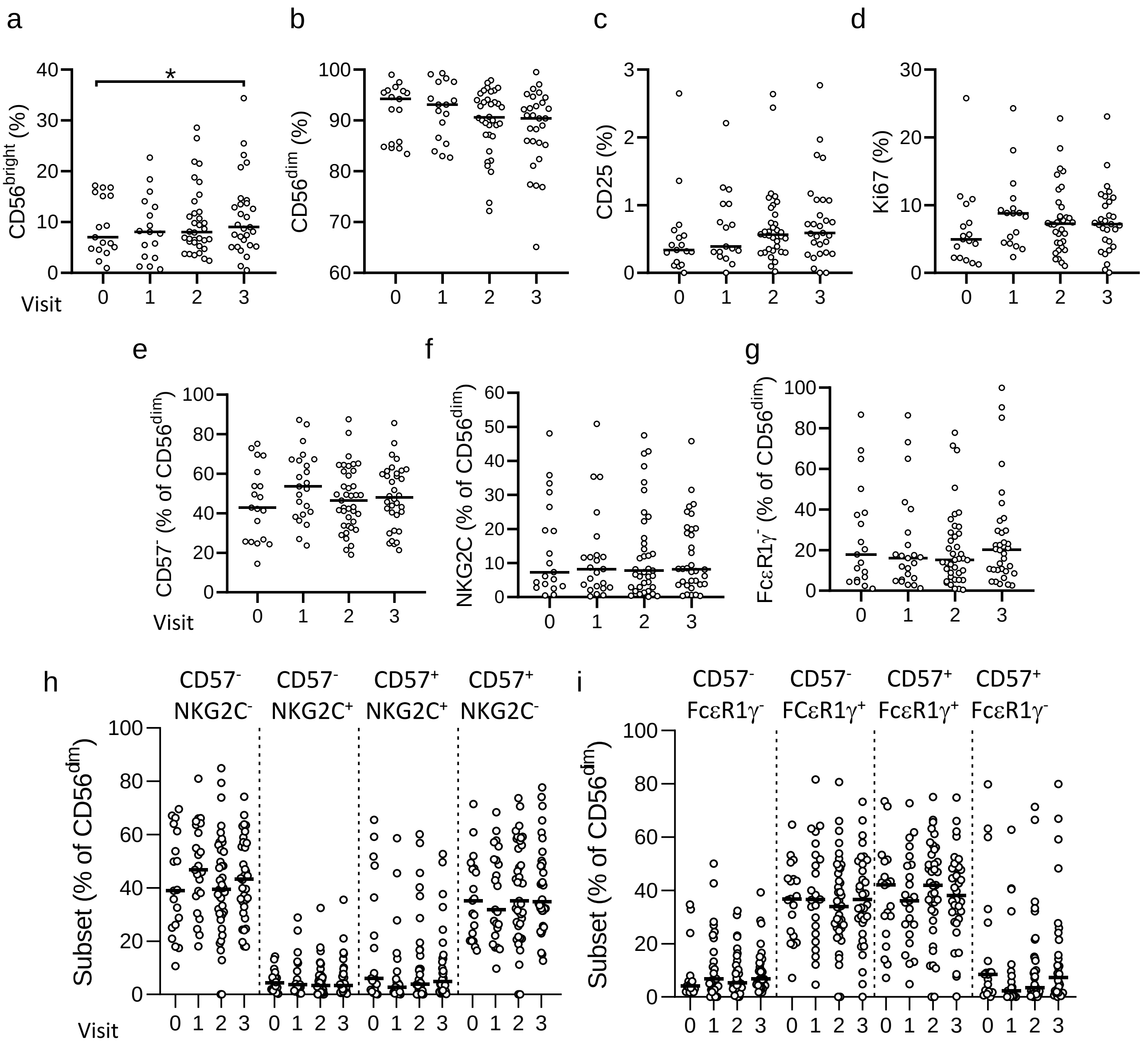

Supplementary Figure 2: Analysis of NK cell *ex vivo* phenotypic data across vaccination visits.

Frequencies of CD56^{bright} (**a**), CD56^{dim} (**b**), CD25 (**c**) and Ki67 (**d**) expressing NK cells and of (e) CD57$^-$ (f) NKG2C$^+$ (g) FcεR1γ$^-$ (h) CD57/NKG2C subsets and (i) CD57/FcεR1γ subsets within CD56^{dim} NK cells before vaccination (visit 0), on day 29, 57 or 85 post-dose 1 (visit 1), on day 14 post-dose 2 (visit 2) and on day 180 post-dose 2 (visit 3) (see Table 1 for n). Data points are shown with bars representing median values. Comparisons across visits were performed one-way ANOVA mixed effects analysis with Geisser-Greenhouse correction. * $p<0.05$.

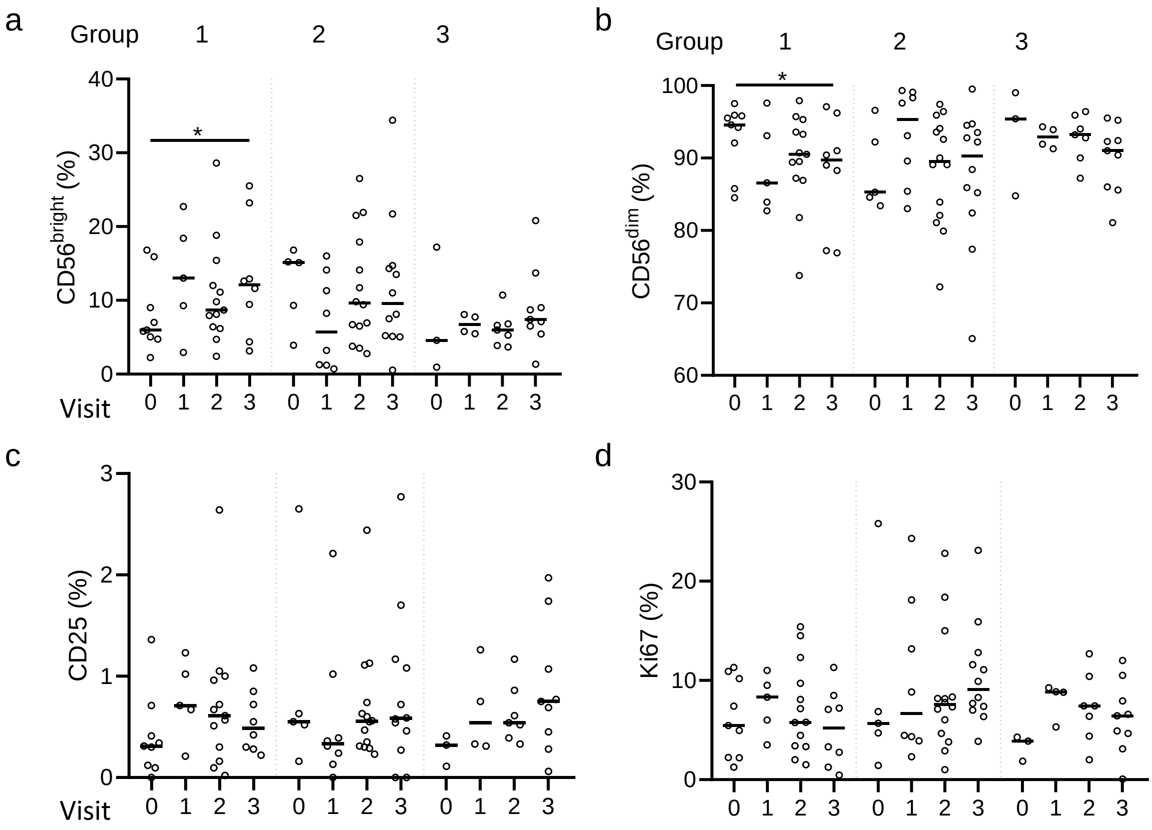

Supplementary Figure 3: Analysis of NK cell *ex vivo* phenotypic data between vaccine study group.

Frequencies of CD56^{bright} (**a**), CD56^{dim} (**b**), CD25 (**c**) and Ki67 (**d**) positive NK cells at each visit according to vaccine study group (groups 1, 2 and 3 received Ad26.ZEBOV on day 1 and MVA-BN-Filo on day 29, 57 or 85 respectively). Data points are shown with bars representing median values. Comparisons across visits were performed one-way ANOVA mixed effects analysis with Geisser-Greenhouse correction. * $p<0.05$.

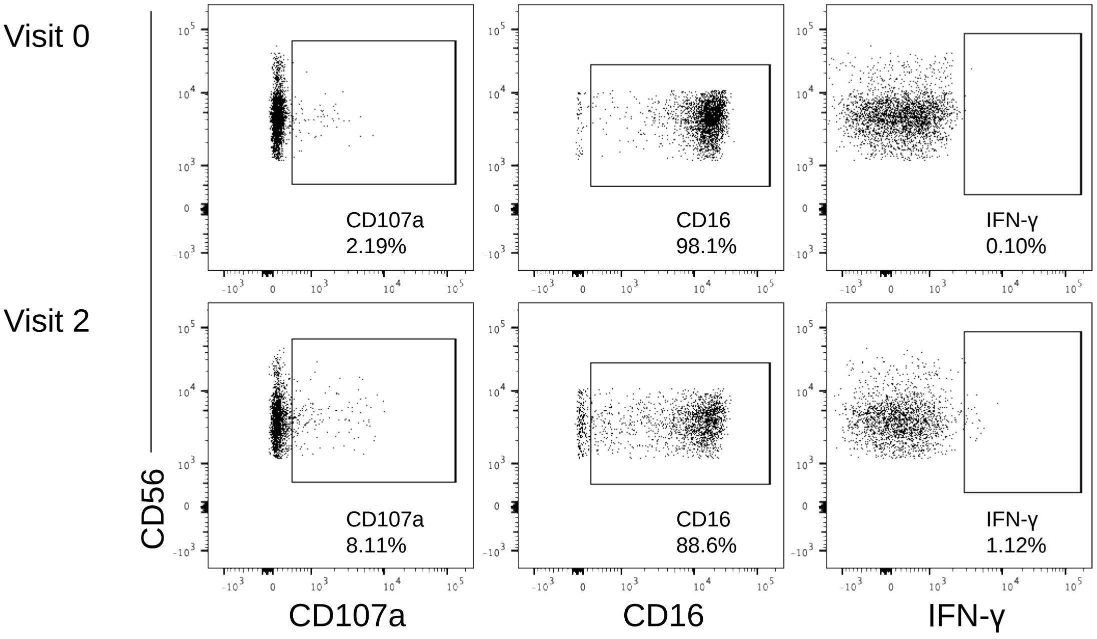

Supplementary Figure 4:
Flow cytometry gating strategy for *in vitro* NK cell activation analysis.
Plots show single, live, $CD56^{+}CD3^{-}$ NK cell CD107a, CD16 and IFN-γ gated following 6 hours *in vitro* culture with plate immobilised EBOV GP and pre or post-vaccination serum (serum from visit 0 and visit 2 are shown as an example). Plots shows PBMC from one non-vaccinated donor.
