# Supplementary material for: Durable natural killer cell responses after heterologous two-dose Ebola vaccination
Source: NPJ Vaccines. 2021 Jan 29;6:19. doi: 10.1038/s41541-021-00280-0 (PMC7846750; doi:10.1038/s41541-021-00280-0)
Supplement: Supplementary file 2 — Reporting Summary [file 41541_2021_280_MOESM2_ESM.pdf]

nature research

Corresponding author(s): DBPR NPJVACCINES-00846R

Last updated by author(s): Nov 13, 2020

Reporting Summary

Nature Research wishes to improve the reproducibility of the work that we publish. This form provides structure for consistency and transparency in reporting. For further information on Nature Research policies, see our Editorial Policies and the Editorial Policy Checklist.

Statistics

For all statistical analyses, confirm that the following items are present in the figure legend, table legend, main text, or Methods section.

n/a	Confirmed	
☐	☒	The exact sample size (*n*) for each experimental group/condition, given as a discrete number and unit of measurement
☐	☒	A statement on whether measurements were taken from distinct samples or whether the same sample was measured repeatedly
☐	☐	The statistical test(s) used AND whether they are one- or two-sided<br>*Only common tests should be described solely by name; describe more complex techniques in the Methods section.*
☐	☒	A description of all covariates tested
☐	☒	A description of any assumptions or corrections, such as tests of normality and adjustment for multiple comparisons
☐	☒	A full description of the statistical parameters including central tendency (e.g. means) or other basic estimates (e.g. regression coefficient) AND variation (e.g. standard deviation) or associated estimates of uncertainty (e.g. confidence intervals)
☒	☐	For null hypothesis testing, the test statistic (e.g. *F*, *t*, *r*) with confidence intervals, effect sizes, degrees of freedom and *P* value noted<br>*Give P values as exact values whenever suitable.*
☒	☐	For Bayesian analysis, information on the choice of priors and Markov chain Monte Carlo settings
☒	☐	For hierarchical and complex designs, identification of the appropriate level for tests and full reporting of outcomes
☒	☐	Estimates of effect sizes (e.g. Cohen's *d*, Pearson's *r*), indicating how they were calculated

Our web collection on statistics for biologists contains articles on many of the points above.

Software and code

Policy information about availability of computer code

Data collection	FACS DIVA 8.0 software, (BD Biosciences, Europe)
Data analysis	FlowJo V10 (Tree Star, Oregon, U.S.A). GraphPad Prism version 7.04 (GraphPad, California, U.S.A.).

For manuscripts utilizing custom algorithms or software that are central to the research but not yet described in published literature, software must be made available to editors and reviewers. We strongly encourage code deposition in a community repository (e.g. GitHub). See the Nature Research guidelines for submitting code & software for further information.

Data

Policy information about availability of data

All manuscripts must include a data availability statement. This statement should provide the following information, where applicable:

- Accession codes, unique identifiers, or web links for publicly available datasets
- A list of figures that have associated raw data
- A description of any restrictions on data availability

The raw data supporting the conclusions of this article will be made available by the authors, without undue reservation, to any qualified researcher

Field-specific reporting

Please select the one below that is the best fit for your research. If you are not sure, read the appropriate sections before making your selection.

☒ Life sciences ☐ Behavioural & social sciences ☐ Ecological, evolutionary & environmental sciences

For a reference copy of the document with all sections, see nature.com/documents/nr-reporting-summary-flat.pdf

Life sciences study design

All studies must disclose on these points even when the disclosure is negative.

Sample size	Sample size for the original clinical trail was established. Not all PBMC samples were available form teh original clinical trial. We have performed a post-hoc power calculation based on the PBMC and serum samples made available (Details of sample numbers are provided in table 1 of the manuscript): Based on effect sizes from our previous studies we calculated for ex-vivo phenotypic analysis that with 10 pairs of individuals we had 80% power to detect a 0.5-fold increase between pre and post vaccination time points at p<0.05. For comparison between groups of n=26 vs n=12 individuals in in-vitro analysis, we had 80% power to detect a 0.5-fold difference in peak responses at p<0.05.
Data exclusions	From the samples made available for the original clinical trial none were excluded from the analysis. For Flow cytometry,
Replication	Replication of flow cytomeric data was inherent in the inclusion of core markers in all detection panels. Control stimuli were included in all acquisition batches to ensure consistency of protocol. application.
Randomization	Participant randomization was performed for the original clinical study using a computer-generated block randomization schedule as previously published for a parallel trial (Reference doi: 10.1093/infdis/jiz071). fIn the current post-hoc studies, randomised codes were known to enable matching samples from an individual participant across the vaccination time-course. All samples from each individual participant were run in a single batch run to enxsure
Blinding	Participants and study team members of the original trial were blinded until 21 days post-boost vaccination. For the current post hoc studies, the codified identity of samples for each study group were known to enable experiments were performed in batches where that an equal number of individuals participants from each group were tested simultaneously in each assay run.

Reporting for specific materials, systems and methods

We require information from authors about some types of materials, experimental systems and methods used in many studies. Here, indicate whether each material, system or method listed is relevant to your study. If you are not sure if a list item applies to your research, read the appropriate section before selecting a response.

Materials & experimental systems

n/a	Involved in the study	
☐	☒	Antibodies
☒	☐	Eukaryotic cell lines
☒	☐	Palaeontology and archaeology
☒	☐	Animals and other organisms
☐	☒	Human research participants
☒	☐	Clinical data
☒	☐	Dual use research of concern

Methods

n/a	Involved in the study	
☒	☐	ChIP-seq
☐	☒	Flow cytometry
☒	☐	MRI-based neuroimaging

Antibodies

Antibodies used	anti-CD3-V500 (clone UCHT1) (BD Biosciences), anti-CD56-BV605 (clone HCD56), anti-IFN-γ-BV785 (clone 4S.B3), anti-CD25-BV785 (clone BC96) (Biolegend, London, U.K.). Anti-CD16-APC (clone CB16), anti-CD57-e450 (clone TB01), Ki67-PerCP-eFluor710 (clone 20Raj1), anti-NKG2C-PE (clone 134591) (R&D systems) and anti-FcεR1γ- FITC (Millipore, U.K, polyclonal reagent, lot 2823182). Catalogue numbers are accessible via the links in the section below.
Validation	Listing below of antibody, validation use and link to validation data.<br>anti-CD3-V500 (clone UCHT1) , mouse anti human: flow cytometry. www.bdbiosciences.com<br>anti-CD56-BV605 (clone HCD56), mouse anti-human: flow cytometry. www.biolegend.com<br>anti-IFN-γ-BV785 (clone 4S.B3),mouse anti-human: flow cytometry. www.biolegend.com<br>anti-CD25-BV785 (clone BC96), mouse anti-human: flow cytometry.www.biolegend.com<br>anti-CD16-APC (clone CB16), mouse anti-human: flow cytometry. www.bdbiosciences.com<br>anti-CD57-e450 (clone TB01), mouse anti-human: flow cytometry. https://www.thermofisher.com<br>anti-Ki67-PerCP-eFluor710 (clone 20Raj1): mouse anti-human: flow cytometry. https://www.thermofisher.com<br>anti-NKG2C-PE (clone 134591) mouse anti-human: flow cytometry. www.rndsystems.com<br>anti-FcεR1γ- FITC: rabbit polyclonal reagent: flow cytometry. www.merckmillipore.com

Human research participants

Policy information about studies involving human research participants

Population characteristics	For the Original clinical trial, eigible, healthy adult volunteers were recruited into a multi-centre, randomised, placebo-controlled, observer blind Ebola vaccine trial, held in Europe; EBL2001 (ClinicalTrials.gov Identifier NCT02416453; registered 15th April 2015, https://clinicaltrials.gov/ct2/show/NCT02416453?term=VAC52150EBL2001&draw=2&rank=2).
Recruitment	For the original study: Eligible participants were healthy men and women aged 18 to 50 years who provided formal,written consent and reported no prior immunization with a candidate Ebola vaccine or any MVA- or Ad26-vectored vaccine.
Ethics oversight	The EBL2001 trial protocol and study documents were approved in France by the French national Ethics Committee "CPP Ile de France III" (reference number 3287) and the French Medicine Agency (ANSM) (reference number 150646A-61) and in the UK by the Medicines and Healthcare Products Regulatory Agency (MHRA) and National Research Ethics Service (Reference South Central – Oxford A 15/SC/0211) and the LSHTM Research Ethics Committee (reference number 14760).

Note that full information on the approval of the study protocol must also be provided in the manuscript.

Flow Cytometry

Plots

Confirm that:

☒ The axis labels state the marker and fluorochrome used (e.g. CD4-FITC).

☒ The axis scales are clearly visible. Include numbers along axes only for bottom left plot of group (a 'group' is an analysis of identical markers).

☒ All plots are contour plots with outliers or pseudocolor plots.

☒ A numerical value for number of cells or percentage (with statistics) is provided.

Methodology

Sample preparation	PBMC from non-vaccinated non-trial volunteers were isolated using Histopaque 1077 gradient centrifugation and cryopreserved in liquid nitrogen or used immediately. PBMC from EBL2001 volunteers from France were isolated using Leucosep tubes, cryopreserved in liquid nitrogen and shipped to LSHTM. Anti-CD107a-FITC (clone H4A3; BD Biosciences) was added to the cultures (2µl per well) for the entire culture period. Cells were stained for all other surface markers including a viability marker (Fixable Viability Stain 700; BD Biosciences) in FACS buffer (PBS, 0.5% FCS, 0.05% sodium azide and 2mM EDTA) for 30 minutes in 96-well round bottom plates after blocking Fc receptors for 5 minutes with Fc Receptor (FcR) Blocking Reagent (Miltenyi Biotec). Cells were then washed in FACS buffer, fixed and permeabilised using Cytofix/Cytoperm Kit (BD Biosciences) or Foxp3/Transcription Factor Fixation/Permeabilisation Kit (eBiosciences) according to the manufacturer's instructions. Cells were then stained for intracellular markers with FcR blocking for 20 minutes and washed again. Finally, cells were resuspended in FACS buffer and analysed.
Instrument	BD LSRII flow cytometer.
Software	FACSDiva 8 software (Becton Dickinson, Europe) and data were analysed using FlowJo V10 (Tree Star, Oregon, U.S.A).
Cell population abundance	No purifications involved: Samples with less than 100 NK cell events were excluded from the analysis (less than 3% of samples).
Gating strategy	FACS gates were set using unstimulated cells or FMO controls. Gating strategies for ex-vivo and post -culture in-vitro analysis analysis are shown in supplementary information

☒ Tick this box to confirm that a figure exemplifying the gating strategy is provided in the Supplementary Information.
